# Association of white matter hyperintensity burden and infarct volume in the anterior choroidal artery territory with early neurological progression: a dual-center retrospective study

**DOI:** 10.3389/fnagi.2025.1577742

**Published:** 2025-05-19

**Authors:** Weiwei Gao, Lixue Wang, Junyi Huang, Yitao Yu, Jingjing She, Mingyang Wang, Lijuan Cai, Taishan Kang, Xingyu Chen, Jianzhong Lin, Renjing Zhu

**Affiliations:** ^1^Department of Neurology, Zhongshan Hospital of Xiamen University, School of Medicine, Xiamen University, National Advanced Center for Stroke, Xiamen, China; ^2^Xiamen Clinical Research Center for Cerebrovascular Diseases, Xiamen, China; ^3^Department of Neurology, Jimusaer County People’s Hospital, Xinjiang, China; ^4^Department of Brain Sciences, Imperial College London, London, United Kingdom; ^5^School of Basic Medical Sciences, Tianjin Medical University, Tianjin, China; ^6^The School of Clinical Medicine, Fujian Medical University, Fuzhou, Fujian, China; ^7^Department of MRI, Zhongshan Hospital of Xiamen University, School of Medicine, Xiamen University, Xiamen, China

**Keywords:** anterior choroidal artery, infarction, early neurological progression, white matter hyperintensity, infarct volume

## Abstract

**Objective:**

To investigate the associations of white matter hyperintensity (WMH) burden and infarct volume with early neurological progression in anterior choroidal artery (AChA) territory infarction, and to identify potential imaging-based predictive thresholds.

**Methods:**

This retrospective cohort study consecutively enrolled AChA infarct patients admitted to two comprehensive stroke centers between September 2018 and September 2024. WMH burden and infarct volume were assessed using the Fazekas visual rating scale and an automated volumetric quantification method based on lesion prediction algorithm, respectively. The primary outcome was early neurological progression. Multivariate logistic regression models with stepwise adjustment for confounders were used to evaluate the associations of WMH burden and infarct volume with early progression. Restricted cubic spline regression was performed to explore non-linear relationships and to determine thresholds. Continuous variables were standardized, and piecewise regression analysis was conducted based on the identified thresholds. Subgroup analyses with interaction tests were performed to assess the consistency of these associations across different populations.

**Results:**

A total of 216 patients were included, of whom 82 (38.0%) experienced early neurological progression. After adjustment for potential confounders, WMH burden showed a significant non-linear association with progression risk. For WMH volumes <66.1 mL, each standard deviation increase was associated with a 74% higher risk of progression (standardized OR: 1.74, 95% CI: 1.29–2.40, *p* < 0.001). Compared with the lowest quartile, patients in the highest WMH quartile showed significantly increased risk (adjusted OR: 5.32, 95% CI: 1.48–13.88, *p* = 0.009). This association was confirmed by Fazekas scale analysis, with grade 3 patients showing substantially higher risk than grade 0 (adjusted OR: 6.22, 95% CI: 1.74–25.42, *p* = 0.007). Infarct volume demonstrated a similar non-linear pattern; for volumes <1.1 mL, each standard deviation increase was associated with 59% higher progression risk (standardized OR: 1.59, 95% CI: 1.04–2.47, *p* = 0.036). Quartile analysis revealed the highest risk in the third quartile compared to the lowest (adjusted OR: 5.63, 95% CI: 2.06–15.40, *p* < 0.001).

**Conclusion:**

This study revealed non-linear associations of WMH and infarct volume with early progression in AChA infarct patients.

## 1 Introduction

The anterior choroidal artery (AChA) is a significant branch arising from the supraclinoid segment of the posterior lateral wall of the internal carotid artery. It supplies critical functional regions including the posterior limb of the internal capsule, cerebral peduncle, lateral thalamus, lateral geniculate body, medial temporal lobe, and hippocampal region (Antoniello et al., 2023; Herman et al., 1966). Although AChA territory infarctions account for only 3.9–8.3% of ischemic strokes, they frequently cause severe motor, sensory, and visual deficits due to their strategic location along crucial white matter tracts (Chausson et al., 2014; Ois et al., 2009; Zhao et al., 2023). More concerning is that over 50% of patients with AChA infarction experience early neurological progression, significantly affecting functional outcomes and long-term prognosis (Derflinger et al., 2015; Ois et al., 2009; Zhao et al., 2023).

Early neurological progression, defined as neurological deterioration during the acute phase of stroke, represents a critical therapeutic window where timely intervention may mitigate permanent tissue damage. Traditional pathophysiological mechanisms of early neurological progression in acute ischemic stroke include hemodynamic abnormalities, microemboli, inflammatory cascades, and blood–brain barrier disruption (Seners et al., 2015). In AChA territory infarction, which commonly manifests as penetrating artery atherosclerotic disease, specific mechanisms may additionally involve parent artery atherosclerotic plaque occluding or extending into the penetrating artery orifice, atherosclerotic plaque at the orifice of penetrating arteries causing luminal occlusion, and unstable plaque detachment leading to distal vessel occlusion (Deguchi and Takahashi, 2023). These acute pathological processes trigger a complex series of cellular and molecular events, ultimately resulting in expansion of the ischemic penumbra, irreversible transformation of salvageable penumbral tissue into infarct core, or induction of serious complications such as cerebral edema, exacerbating neurological impairment (Shkirkova et al., 2018).

Concurrently, white matter hyperintensities (WMH), appearing as enhanced signal on T2-weighted magnetic resonance imaging, represent an important neuroimaging marker of chronic cerebral small vessel disease. In populations over 40 years of age, WMH prevalence reaches 36.5–100% (Huang et al., 2024), reflecting cumulative microvascular damage resulting from various pathophysiological processes including chronic hypoperfusion, decreased cerebrovascular reactivity, blood–brain barrier dysfunction, oxidative stress, and neuroinflammation (Wu et al., 2021). The temporal evolution of WMH spans years to decades, contrasting sharply with the acute timeline of neurological progression measured in hours to days.

Despite these differing temporal characteristics, compelling evidence suggests a potential close association between chronic WMH burden and acute neurological deterioration events. Multiple studies have investigated the association between WMH burden and early neurological progression in various stroke subtypes (including isolated pontine infarction and cryptogenic stroke), with inconsistent results. Some studies indicate that WMH significantly increases the risk of early progression (Nam et al., 2016; Wang et al., 2023; Zhang L. et al., 2024; Zhang W. et al., 2024; Zhou et al., 2020), while others found no significant association (Chen et al., 2017; Song et al., 2024). These discrepancies may stem from the heterogeneity of stroke populations, variations in neurological progression definitions, and methodological limitations in WMH assessment. Most previous studies employed semi-quantitative visual rating scales (such as the Fazekas scale) to evaluate WMH, potentially underestimating the true strength of the association due to inability to capture the spatial heterogeneity and volumetric burden of white matter damage. Similarly, infarct size has been identified as another key imaging predictor of stroke outcomes. Research suggests that AChA infarct size positively correlates with the degree of neurological deficit, with larger lesions closely associated with higher risk of poor prognosis (Chausson et al., 2014; Derflinger et al., 2015; Ois et al., 2009; Zhao et al., 2023). However, most previous studies used maximal lesion diameter as the assessment metric, which fails to comprehensively reflect the actual spatial distribution and extent of tissue damage. The lack of precise quantitative analysis of infarct volume may have limited accurate assessment of its predictive value (Yang et al., 2023).

Despite the high incidence of early progression in patients with AChA territory infarction, the specific relationship between WMH burden, infarct volume, and early neurological progression remains unexplored. Understanding this relationship could significantly enhance risk stratification capabilities and guide individualized monitoring and intervention strategies, potentially improving prognosis in this vulnerable population.

Therefore, this study aims to systematically investigate the association between WMH burden, infarct volume in the anterior choroidal artery territory, and early neurological progression using a combination of quantitative volumetric measurements and visual rating scales. Specifically, we seek to determine whether these imaging markers exhibit linear or non-linear relationships with progression risk, identify threshold effects that may aid clinical decision-making, and explore the consistency of these associations across different population subgroups. Through this comprehensive approach, we hope to establish more reliable imaging biomarkers for early risk stratification in patients with AChA territory infarction.

## 2 Materials and methods

### 2.1 Study design

This study was a dual-center retrospective cohort study that consecutively enrolled patients with AChA territory infarction admitted to two comprehensive stroke centers (Zhongshan Hospital of Xiamen University and Jimusaer County People’s Hospital) between September 2018 and September 2024. The inclusion criteria were: (1) age ≥18 years; (2) time from symptom onset to admission <7 days; (3) acute ischemic lesions in the AChA territory confirmed by diffusion-weighted imaging (DWI); (4) modified Rankin Scale (mRS) score ≤2 before stroke onset. The exclusion criteria were: (1) presence of acute infarct lesions in other arterial territories on DWI; (2) pre-existing neurological diseases that may affect white matter assessment, including traumatic brain injury, brain tumors, and infectious, neuroimmunological, toxic, or metabolic encephalopathies; (3) history of stroke with significant sequelae, or mild sequelae similar to the current clinical presentation that may affect assessment; and (4) incomplete clinical data. This study was approved by the ethics committees of both participating hospitals, and written informed consent was obtained from all participants or their legal representatives.

The imaging diagnostic criteria for AChA territory infarction (Antoniello et al., 2023; Herman et al., 1966; Hupperts et al., 1994) were defined as follows: the main body of the infarct lesion (>2/3 of the volume) was located within the probability distribution of the AChA territory, with typical distribution patterns accessible at https://stroke-maps.github.io/anterior-choroidal. The infarct involved one or more of the following anatomical structures: posterior 2/3 of the posterior limb of the internal capsule, middle and posterior periventricular region (corona radiata), lateral thalamus, lateral geniculate body, medial globus pallidus, tail of the caudate nucleus, medial temporal lobe, hippocampal region, and basal 1/3 of the cerebral peduncle. Patients with any of the following conditions were not included in this study: (1) simultaneous presence of hemispheric infarction in the posterior limb of the internal capsule or periventricular region; (2) periventricular infarction extending beyond the posterior region (such as long-segment periventricular infarction or involvement of the subcortical region); and (3) simultaneous presence of basal ganglia infarction in the posterior limb of the internal capsule, with large putaminal infarction not limited to the posteromedial part. All imaging diagnoses were made jointly by a senior neurologist and a senior radiologist. In case of diagnostic discrepancies, a third physician was involved in the discussion to reach a consensus.

### 2.2 Data collection

Clinical data of all enrolled patients were systematically collected through the electronic medical record system, including demographic information (age, sex), vascular risk factors [hypertension, diabetes mellitus, hyperlipidemia, coronary artery disease, atrial fibrillation, history of stroke/transient ischemic attack (TIA)], lifestyle factors (current smoker, alcohol consumption), vital signs on admission (systolic blood pressure, diastolic blood pressure), and neurological function assessments [National Institutes of Health Stroke Scale score (NIHSS), mRS]. For vascular morphology assessment, we focused on examining the ipsilateral carotid artery of the infarcted side. Carotid stenosis was evaluated using Carotid Doppler Ultrasonography, with stenosis ≥50% defined as moderate-to-severe stenosis. Additionally, we collected computed tomographic angiography (CTA) results of the head and neck vessels from the affected side. According to the North American Symptomatic Carotid Endarterectomy Trial criteria, carotid stenosis was classified into three grades: mild (0–49%), moderate (50–69%), and severe (70–99%).

Laboratory test indicators included blood cell analysis (neutrophils, lymphocytes, monocytes, red blood cell count, hemoglobin, platelet count), biochemical tests [total protein, albumin, triglycerides, total cholesterol, low-density lipoprotein cholesterol (LDL-C), blood urea nitrogen, creatinine, potassium, fasting blood glucose], and coagulation function (fibrinogen). Treatment plans, including intravenous thrombolysis and antiplatelet therapy strategies, were also recorded. Antiplatelet therapy was divided into three regimens: no antiplatelet therapy, single antiplatelet therapy (aspirin 100 mg/d or clopidogrel 75 mg/d), and dual antiplatelet therapy (aspirin 100 mg/d combined with clopidogrel 75 mg/d).

### 2.3 Imaging analysis

All patients underwent 1.5T or 3.0T cranial magnetic resonance imaging (MRI) scans (Siemens Healthcare, Erlangen, Germany) within 24 h of admission. Standardized scanning sequences included T1-weighted imaging (T1WI), T2-weighted imaging (T2WI), fluid-attenuated inversion recovery (FLAIR), and DWI. To ensure data consistency between the two centers, FLAIR and DWI sequences were acquired using uniform scanning parameters. The FLAIR scanning parameters were: TR 7800 ms, TE 89 ms, IR 2500 ms, FOV 256 mm × 256 mm, matrix 256 × 256, slice thickness 5.0 mm, interslice gap 1.5 mm, and 40 slices. The DWI scanning parameters were: TR 3400 ms, TE 105 ms, FOV 160 mm × 160 mm, matrix 160 × 160, slice thickness 5.0 mm, interslice gap 1.5 mm, and 40 slices. Imaging analysis was performed independently by two experienced neuroradiologists (both with >10 years of experience in stroke imaging diagnosis). The size of acute infarct lesions was assessed based on the maximum diameter on axial DWI, and lesions were classified as small (<20 mm) or large (≥20 mm).

Quantitative analysis of WMH volume was performed using an automated segmentation and semi-automated DWI volumetric segmentation process for acute cerebral infarction: (1) Image preprocessing: DICOM images were converted to NIfTI format using the dcm2niix command; (2) Lesion segmentation: Based on the MATLAB platform, the lesion prediction algorithm (LPA) in the Lesion Segmentation Toolbox (LST) was used to automatically segment WMH lesions on FLAIR images, while a semi-automated threshold method was used to segment acute infarct lesions on DWI images; (3) Spatial normalization and volume calculation: First, FLAIR, DWI, and ADC images were rigidly registered to the T1WI of the same patient using the FLIRT tool in the FSL software package. Next, a non-linear registration algorithm was used to register individual T1WI to the MNI152_T1_2mm standard brain template space. Finally, the transformation matrices obtained from the above registration processes were applied to the WMH and infarct lesion segmentation results, mapping them to the standard space and calculating lesion volumes.

#### 2.3.1 Qualitative assessment of WMH burden was performed using the Fazekas visual rating scale

Based on the location of lesions relative to the lateral ventricles, WMHs were classified as periventricular white matter hyperintensities (PVWMH) and deep white matter hyperintensities (DWMH). The PVWMH scoring criteria were: 0 = no lesions; 1 = cap-shaped or pencil-thin lining; 2 = smooth halo; 3 = irregular periventricular hyperintensities extending into the deep white matter. The DWMH scoring criteria were: 0 = no lesions; 1 = punctate foci; 2 = beginning confluence of foci; 3 = large confluent areas. The total Fazekas score was the sum of the PVWMH and DWMH scores, and the severity of WMHs was classified as no lesions (0), mild (1–2), moderate (3–4), and severe (5–6).

### 2.4 Definition of early neurological progression

Unlike general ischemic stroke, early neurological progression in patients with AChA territory infarction has certain unique features. The traditional definition (an increase of ≥2 points in the NIHSS score or ≥1 point in the motor function score) may not be suitable for patients with AChA territory infarction, mainly because these patients often have lower baseline NIHSS scores, and the assessment items are primarily focused on motor function (Liu et al., 2023). Therefore, this study adopted a more sensitive definition of early neurological progression: any persistent neurological worsening after symptom onset was considered early progression, except for those who rapidly recovered after brief fluctuations. In addition, a history of symptom worsening before hospital admission was also considered early progression (Derflinger et al., 2015).

### 2.5 Statistical analysis

Statistical analysis was performed using R software (Version 4.2.2, R Foundation for Statistical Computing, Vienna, Austria). The Shapiro–Wilk test was used to assess the normality of continuous variables. Normally distributed continuous variables were expressed as mean ± standard deviation and compared between groups using the independent samples *t*-test. Non-normally distributed continuous variables were expressed as median (interquartile range) and compared using the Mann–Whitney *U* test. Categorical variables were expressed as frequencies (percentages) and analyzed using Pearson’s chi-square test or Fisher’s exact test.

To investigate the association between WMH burden, infarct volume, and early neurological progression, we constructed three progressively adjusted multivariable logistic regression models. Variable selection followed a stepwise approach: first, we conducted univariate analyses and included covariates with statistical significance (*p* < 0.05) as candidate variables; second, we assessed multicollinearity among these candidate variables by calculating variance inflation factors (VIF) and tolerance values, excluding highly correlated variables based on predetermined criteria (VIF < 10 and tolerance>0.1) (Supplementary Table 1). Model 1 was unadjusted; Model 2 was adjusted for age, diabetes mellitus, baseline NIHSS score, baseline mRS score, lesion size, and affected anatomical sites (lateral thalamus, lateral geniculate body, medial temporal lobe/hippocampus); Model 3 further adjusted for laboratory indicators (neutrophil count, LDL-C, fibrinogen) based on Model 2.

Restricted cubic spline (RCS) regression models were used to assess possible non-linear associations between changes in WMH burden and infarct volume with progression. We set four knots at the 5th, 35th, 65th, and 95th percentiles to construct the RCS functions. After determining the thresholds, continuous variables were standardized, and piecewise regression analysis was performed based on the thresholds. Additionally, we conducted subgroup analyses stratified by sex, age (<60 years vs. ≥60 years), vascular risk factors (hypertension, diabetes, hyperlipidemia, coronary heart disease, atrial fibrillation), and lifestyle factors (smoking, alcohol consumption). Interaction terms were used to test the heterogeneity of effects between subgroups. Variables with statistical significance (*p* < 0.05) in univariate analysis were included as covariates in all analyses. To ensure model robustness, we excluded the lateral geniculate nucleus subgroup from the analysis due to its small sample size (*N* = 4) and zero events in the non-progression group. All statistical tests were two-sided, and a *p*-value <0.05 was considered statistically significant.

## 3 Results

### 3.1 Demographic and clinical characteristics

**Figure 1:**
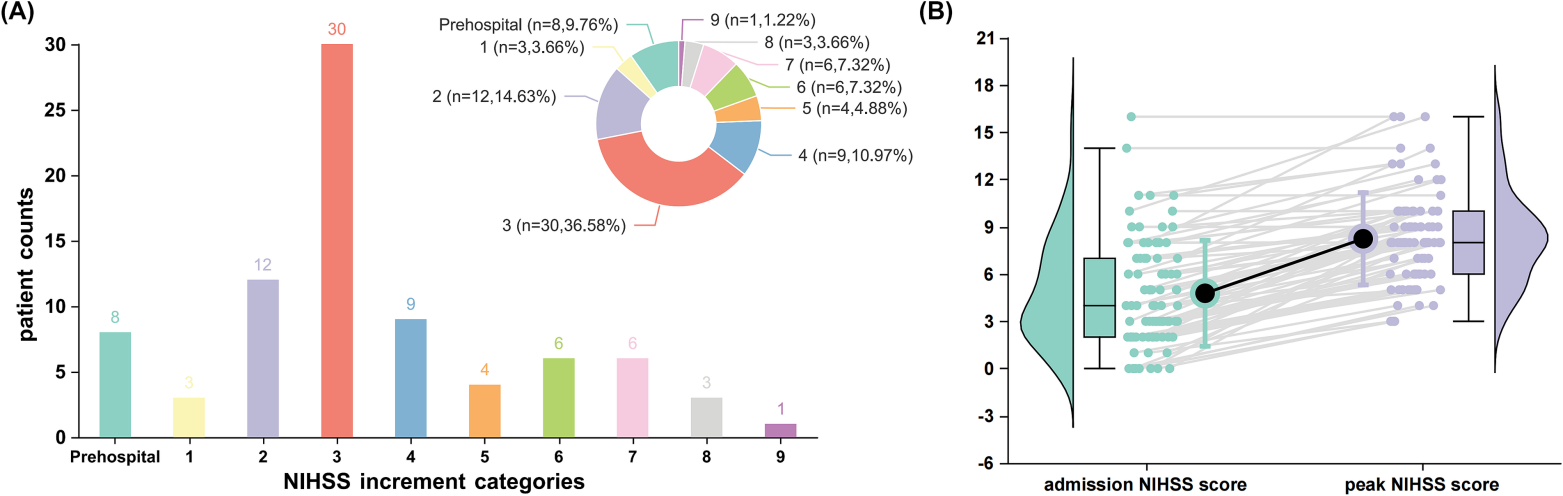
Patterns of early neurological deterioration in patients with anterior choroidal artery territory infarction. **(A)** Distribution of National Institutes of Health Stroke Scale (NIHSS) score increments among patients experiencing early neurological deterioration (*n* = 82, representing 38.0% of the total cohort). The bar chart and corresponding pie chart illustrate the frequency and proportion of patients across different NIHSS increment categories. Notably, 8 patients (9.76%) exhibited prehospital deterioration, while the highest proportion (30 patients [36.58%]) demonstrated an increase of ≥3 points in NIHSS score during hospitalization. **(B)** Comparative analysis of admission versus peak NIHSS scores in patients with early neurological deterioration. Box plots (showing median, interquartile range, and range) with corresponding violin plots display the distribution of scores at each time point. Individual patient trajectories are represented by gray lines connecting paired measurements. Black circles represent mean NIHSS scores.

**Figure 2:**
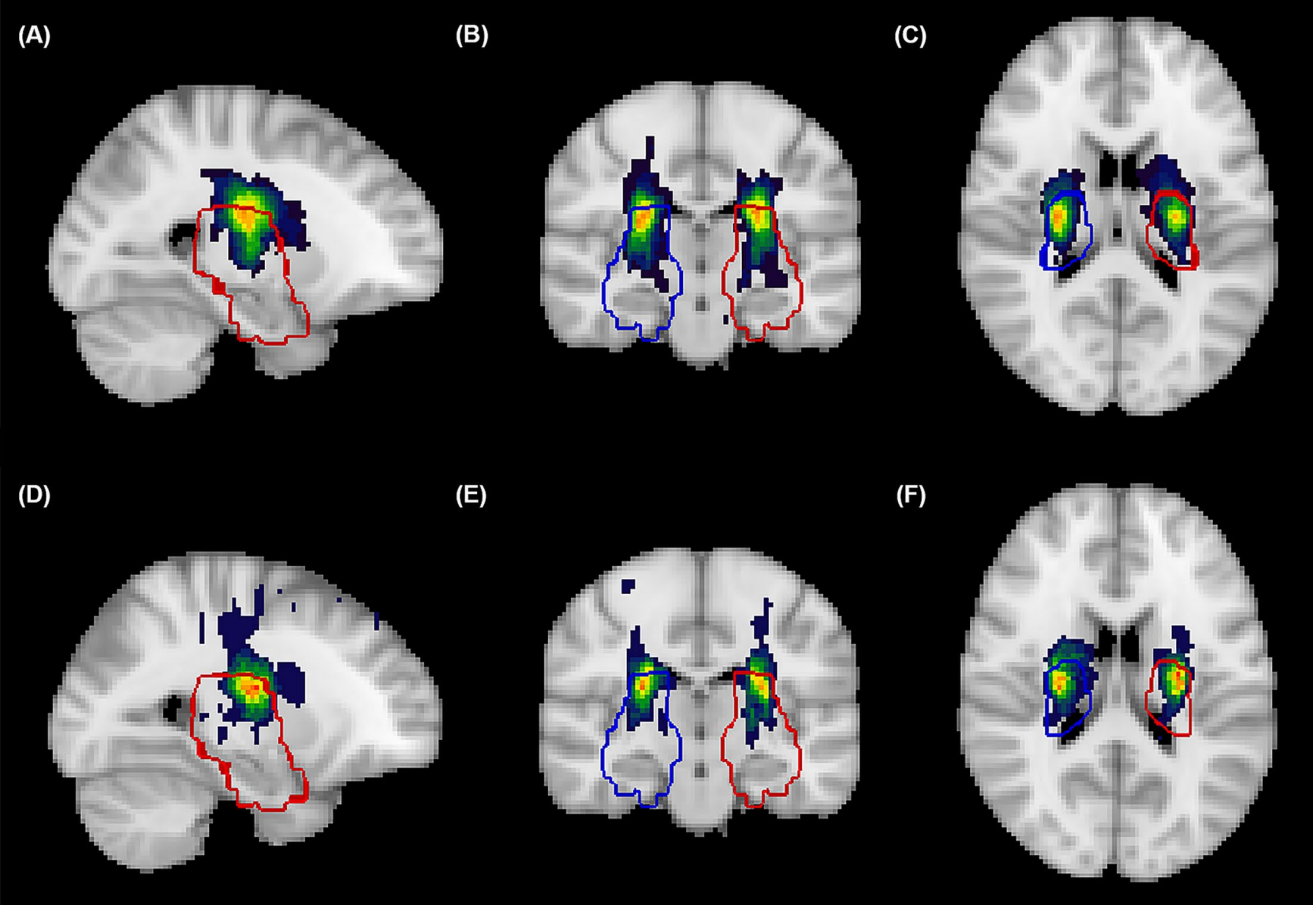
Spatial probability maps of acute infarcts in the anterior choroidal artery territory. Probabilistic heat maps in standardized MNI152 space displaying the cumulative spatial distribution of Anterior Choroidal Artery (AChA) territory acute infarcts from patients at the Xiamen center **(A–C)** and Xinjiang center **(D–F)**, visualized in sagittal **(A,D)**, coronal **(B,E)**, and axial **(C,F)** planes. Color intensity represents infarction frequency, with warmer colors (red-blue) indicating regions with higher probability of infarction. Blue contours delineate the right hemisphere AChA territory, while red contours delineate the left hemisphere AChA territory, both derived from established vascular templates (https://stroke-maps.github.io/anterior-choroidal). The probability maps demonstrate predominant involvement of the posterior limb of internal capsule, periventricular white matter, lateral thalamus, and medial temporal regions, with subtle distributional variations between centers. All lesions were confirmed on diffusion-weighted imaging and spatially normalized to standard space using non-linear transformation for group-level analysis.

A total of 216 patients with AChA territory infarction were included in this study, with the distribution of AChA infarct lesions from both centers illustrated in Figure 1. Among these patients, 82 (38.0%) experienced early neurological progression. Figure 2 details the difference between peak NIHSS scores and admission NIHSS scores; notably, 8 patients had already experienced neurological deterioration prior to admission, while 30 patients exhibited an increase in NIHSS score ≥3 points during hospitalization.

Patients in the progression group were significantly older than those in the non-progression group (66 ± 12 years vs. 61 ± 14 years, *p* = 0.013). The baseline NIHSS score [4.0 (2.0–7.0) vs. 3.0 (2.0–5.0), *p* = 0.023] and mRS score [3.00 (1.00–4.00) vs. 2.00 (1.00–4.00), *p* < 0.001] were also significantly higher in the progression group. Regarding vascular risk factors, the proportion of patients with diabetes mellitus was significantly higher in the progression group than in the non-progression group (32.93% vs. 20.15%, *p* = 0.035) (Table 1).

**Table 1:** Baseline characteristics of patients with anterior choroidal artery territory infarction stratified by early neurological progression.

Variables	Overall *N* = 216	Non-progression *N* = 134	Progression *N* = 82	*p*-value
Age, years	63 ± 13	61 ± 14	66 ± 12	**0.013**
Sex, male, *n* (%)	155 (71.76)	99 (73.88)	56 (68.29)	0.376
Current smoker, *n* (%)	77 (35.65)	51 (38.06)	26 (31.71)	0.344
Alcohol consumption, *n* (%)	42 (19.44)	23 (17.16)	19 (23.17)	0.279
Medical history, *n* (%)				
Hypertension	164 (75.93)	98 (73.13)	66 (80.49)	0.220
Diabetes mellitus	54 (25.00)	27 (20.15)	27 (32.93)	**0.035**
Hyperlipidemia	78 (36.11)	47 (35.07)	31 (37.80)	0.685
Atrial fibrillation	4 (1.85)	2 (1.49)	2 (2.44)	0.635
Previous stroke/TIA	38 (17.59)	25 (18.66)	13 (15.85)	0.600
Coronary artery disease	19 (8.80)	15 (11.19)	4 (4.88)	0.112
Systolic blood pressure, mmHg	150 (138, 166)	150 (140, 166)	152 (138, 170)	0.801
Diastolic blood pressure, mmHg	90 (81, 99)	91 (80, 99)	90 (81, 100)	0.551
Baseline NIHSS score	3.0 (2.0, 6.0)	3.0 (2.0, 5.0)	4.0 (2.0, 7.0)	**0.023**
Baseline mRS score	2.0 (1.0, 4.0)	2.00 (1.0, 4.0)	3.00 (1.0, 4.0)	**<0.001**
Peak NIHSS score	5.0 (2.0, 8.0)	3.0 (2.0, 5.0)	8.0 (6.0, 10.0)	**<0.001**
Lesion side, *n* (%)				0.219
Left	107 (49.54)	62 (46.27)	45 (54.88)	
Right	109 (50.46)	72 (53.73)	37 (45.12)	
Lesion size, *n* (%)				**0.002**
Small infarcts (<20 mm)	173 (80.09)	116 (86.57)	57 (69.51)	
Large infarcts (≥20 mm)	43 (19.91)	18 (13.43)	25 (30.49)	
Anatomical involvement, *n* (%)				
Posterior limb of internal capsule	170 (78.70)	100 (74.63)	70 (85.37)	0.061
Corona radiata	157 (72.69)	95 (70.90)	62 (75.61)	0.450
Medial globus pallidus	14 (6.48)	8 (5.97)	6 (7.32)	0.696
Tail of caudate nucleus	23 (10.65)	11 (8.21)	12 (14.63)	0.137
Lateral thalamus	25 (11.57)	20 (14.93)	5 (6.10)	**0.049**
Lateral geniculate body	4 (1.85)	0 (0.00)	4 (4.88)	**0.020**
Medial temporal lobe/hippocampus	10 (4.63)	3 (2.24)	7 (8.54)	**0.045**
Cerebral peduncle	3 (1.39)	2 (1.49)	1 (1.22)	>0.99
Ipsilateral ICAS on CTA, *n* (%)				>0.99
Mild (0–49%)	14 (24.56)	7 (25.93)	7 (23.33)	
Moderate to severe (≥50%)	1 (1.75)	0 (0.00)	1 (3.33)	
Ipsilateral MCA stenosis on CTA, *n* (%)				0.195
Mild (0–49%)	5 (8.77)	1 (3.70)	4 (13.33)	
Moderate to severe (≥50%)	5 (8.77)	4 (14.81)	1 (3.33)	
Ipsilateral ICAS on DUS, *n* (%)	2 (1.07)	1 (0.86)	1 (1.41)	>0.99
Intravenous thrombolysis, n (%)	39 (18.06)	28 (20.90)	11 (13.41)	0.165
Antiplatelet therapy, *n* (%)				0.388
No antiplatelet	6 (2.78)	5 (3.73)	1 (1.22)	
Single antiplatelet	74 (34.26)	42 (31.34)	32 (39.02)	
Dual antiplatelet	136 (62.96)	87 (64.93)	49 (59.76)	

Values are presented as *n* (%), mean ± SD, or median (IQR) as appropriate. NIHSS, National Institutes of Health Stroke Scale; mRS, modified Rankin Scale; TIA, transient ischemic attack; IQR, interquartile range; SD, standard deviation; ICAS, intracranial arterial stenosis; CTA, Computed Tomography Angiography; MCA, middle cerebral artery; DUS, Doppler Ultrasonography. Bold *p*-values indicate statistical significance (*p* < 0.05).

In terms of imaging characteristics, the proportion of large lesions (≥20 mm) was significantly higher in the progression group than in the non-progression group (30.49% vs. 13.43%, *p* = 0.002). Regarding the involvement of anatomical structures, there were no significant differences between the two groups in the involvement of the posterior limb of the internal capsule (85.37% vs. 74.63%, *p* = 0.061) and the periventricular corona radiata (75.61% vs. 70.90%, *p* = 0.450). However, the progression group had a significantly higher involvement rate of the lateral geniculate body (4.88% vs. 0.00%, *p* = 0.020) and the medial temporal lobe/hippocampus (8.54% vs. 2.24%, *p* = 0.045), while the involvement rate of the lateral thalamus was significantly lower in the progression group (6.10% vs. 14.93%, *p* = 0.049). For vascular morphological assessment, only 57 patients (26.39%) underwent complete head and neck CTA examination, while 187 patients (86.57%) completed carotid Doppler ultrasonography. No significant differences in the degree of vascular stenosis were observed between the progression and non-progression groups (*p* > 0.05). In terms of treatment strategies, there were no significant differences between the two groups in the use of intravenous thrombolysis or antiplatelet therapy strategies (no antiplatelet therapy, single antiplatelet therapy, and dual antiplatelet therapy). There were no significant differences in other baseline characteristics between the two groups.

### 3.2 Laboratory parameters

Regarding laboratory test indicators (Table 2), the progression group had significantly higher neutrophil counts [4.89 (3.93–6.62) vs. 4.62 (3.35–5.89) × 10^9^/L, *p* = 0.013], LDL-C levels [3.39 (2.90–3.98) vs. 3.07 (2.64–3.61) mmol/L, *p* = 0.004], and fibrinogen levels [3.24 (2.71–3.72) vs. 2.97 (2.45–3.48) g/L, *p* = 0.026] compared to the non-progression group. Fasting blood glucose levels showed an increasing trend in the progression group [6.17 (5.21–7.48) vs. 5.54 (5.08–6.89) mmol/L, *p* = 0.065], but the difference did not reach statistical significance. There were no significant differences in other laboratory indicators between the two groups.

**Table 2:** Laboratory findings of anterior choroidal artery territory infarction patients with and without early neurological progression.

Variables	Overall *N* = 216	No progression *N* = 134	Progression *N* = 82	*P*-value
Neutrophils, ×10^9^/L	4.72 (3.48, 6.11)	4.62 (3.35, 5.89)	4.89 (3.93, 6.62)	**0.013**
Lymphocytes, ×10^9^/L	1.71 (1.33, 2.10)	1.71 (1.41, 2.08)	1.66 (1.23, 2.21)	0.463
Monocytes, ×10^9^/L	0.42 (0.34, 0.55)	0.42 (0.34, 0.54)	0.43 (0.35, 0.56)	0.491
Red blood cells, ×10^12^/L	4.63 (4.32, 4.94)	4.63 (4.31, 4.92)	4.63 (4.42, 4.96)	0.584
Hemoglobin, g/L	143 (131, 151)	144 (133, 151)	142 (130, 150)	0.384
Platelets, ×10^9^/L	231 (190, 268)	228 (189, 260)	232 (191, 269)	0.542
Total protein, g/L	70 (66, 74)	69 (67, 73)	72 (66, 77)	0.057
Albumin, g/L	41.1 ± 3.4	41.3 ± 3.1	40.7 ± 3.7	0.255
Triglycerides, mmol/L	1.35 (0.97, 2.04)	1.35 (1.00, 2.13)	1.36 (0.96, 1.97)	0.498
Total cholesterol, mmol/L	5.10 ± 1.21	5.03 ± 1.11	5.21 ± 1.36	0.328
LDL cholesterol, mmol/L	3.21 (2.70, 3.78)	3.07 (2.64, 3.61)	3.39 (2.90, 3.98)	**0.004**
BUN, mmol/L	5.19 (4.29, 6.38)	5.25 (4.43, 6.40)	4.90 (4.00, 6.36)	0.165
Creatinine, μmol/L	72 (63, 82)	72 (62, 82)	73 (63, 84)	0.563
Potassium, mmol/L	3.85 ± 0.36	3.87 ± 0.35	3.82 ± 0.36	0.349
FBG, mmol/L	5.76 (5.10, 7.01)	5.54 (5.08, 6.89)	6.17 (5.21, 7.48)	0.065
Fibrinogen, g/L	3.06 (2.58, 3.60)	2.97 (2.45, 3.48)	3.24 (2.71, 3.72)	**0.026**

Values are presented as median (IQR) or mean ± SD as appropriate. BUN, blood urea nitrogen; LDL, low-density lipoprotein; FBG, fasting blood glucose; IQR, interquartile range; SD, standard deviation. Bold *p*-values indicate statistical significance (*p* < 0.05).

### 3.3 Associations of WMH and infarct volume with early progression

**Figure 3:**
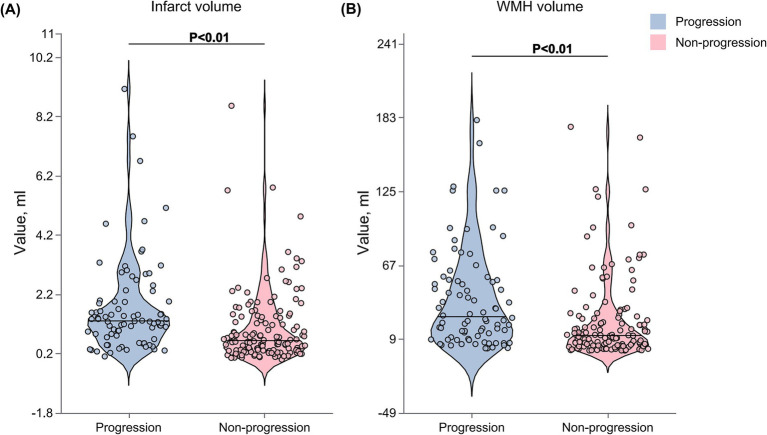
Comparison of WMH and infarct volumes between progression and non-progression groups. Violin plots showing the distribution of WMH and infarct volumes in patients with and without early neurological progression. **(A)** WMH volume was significantly higher in the progression group compared with the non-progression group. **(B)** Similarly, infarct volume was larger in the progression group. The Violin plots display the median (horizontal line within the box), interquartile range (box boundaries), and minimum and maximum values (whiskers). Outliers are represented by individual data points.

**Figure 4:**
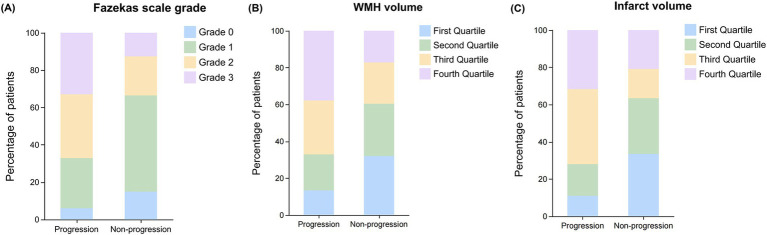
Distribution of Fazekas scale grades, WMH volume, and infarct volume quartile between progression and non-progression groups. Bar graphs showing the distribution patterns of white matter lesion burden and infarct volume in patients with and without early neurological progression. **(A)** Distribution of Fazekas scale grades (0–3) showing a higher proportion of severe lesions (grades 2–3) in the progression group compared to the non-progression group. **(B)** Distribution of WMH volume by quartiles showing increased representation of higher quartiles (third and fourth) in the progression group. **(C)** Distribution of infarct volume by quartiles demonstrating a greater proportion of patients with larger infarct volumes (third and fourth quartiles) in the progression group compared to the non-progression group.

The total WMH volume was significantly larger in the progression group compared to the non-progression group [27 (11–56) vs. 12 (4–27) mL, *p* < 0.001] (Figure 3A). In the quartile analysis of WMH volume (Figure 4B), the proportion of patients in Quartile 4 was significantly higher in the progression group (37.80% vs. 17.16%), while the proportion in the Quartile 1 was significantly lower in the progression group (13.41% vs. 32.09%, *p* < 0.001) (Table 3).

**Table 3:** White matter hyperintensities and infarct volume characteristics in anterior choroidal artery territory infarction patients.

Variables	Overall *N* = 216	Non-progression *N* = 134	Progression *N* = 82	*p*-value
Total WMH volume, mL	16 (6, 42)	12 (4, 27)	27 (11, 56)	<0.001
WMH volume quartiles				<0.001
Quartile 1	54 (25.00)	43 (32.09)	11 (13.41)	
Quartile 2	54 (25.00)	38 (28.36)	16 (19.51)	
Quartile 3	54 (25.00)	31 (22.39)	23 (29.27)	
Quartile 4	54 (25.00)	22 (17.16)	32 (37.80)	
Periventricular WMH score				<0.001
Grade 0	26 (12.04)	23 (17.16)	3 (3.66)	
Grade 1	91 (42.13)	68 (50.75)	23 (28.05)	
Grade 2	55 (25.46)	24 (17.91)	31 (37.80)	
Grade 3	44 (20.37)	19 (14.18)	25 (30.49)	
Deep WMH score				<0.001
Grade 0	76 (35.19)	60 (44.78)	16 (19.51)	
Grade 1	82 (37.96)	51 (38.06)	31 (37.80)	
Grade 2	29 (13.43)	13 (9.70)	16 (19.51)	
Grade 3	29 (13.43)	10 (7.46)	19 (23.17)	
Total Fazekas scale score				<0.001
Grade 0 (none)	25 (11.57)	20 (14.93)	5 (6.10)	
Grade 1 (mild)	91 (42.13)	69 (51.49)	22 (26.83)	
Grade 2 (moderate)	56 (25.93)	28 (20.90)	28 (34.15)	
Grade 3 (severe)	44 (20.37)	17 (12.69)	27 (32.93)	
Infarct volume, mL	0.90 (0.39, 1.63)	0.64 (0.31, 1.46)	1.30 (0.82, 1.98)	<0.001
Infarct volume quartiles				<0.001
Quartile 1	54 (25.00)	45 (33.58)	9 (10.98)	
Quartile 2	54 (25.00)	40 (29.85)	14 (17.07)	
Quartile 3	54 (25.00)	21 (15.67)	33 (40.24)	
Quartile 4	54 (25.00)	28 (20.90)	26 (31.71)	

Values are presented as median (IQR) or *n* (%) as appropriate. WMH, white matter hyperintensities; IQR, interquartile range. WMH quartiles (mL): Q1 [0–5.61], Q2 [5.61–15.9], Q3 [15.9–42], Q4 [42–181.0]. Infarct volume quartiles (mL): Q1 [0–0.387], Q2 [0.387–0.896], Q3 [0.896–1.63], Q4 [1.63–9.14].

In the Fazekas scale assessment (Figure 4A), the PVWMH scores showed that the proportion of severe lesions (Grade 2: 37.80% vs. 17.91%; Grade 3: 30.49% vs. 14.18%) was significantly higher in the progression group (*p* < 0.001). The DWMH scores also showed a similar trend, with the progression group having a significantly higher proportion of severe lesions (Grade 2: 19.51% vs. 9.70%; Grade 3: 23.17% vs. 7.46%) compared to the non-progression group (*p* < 0.001). The results of the total Fazekas score were consistent, with a significantly increased proportion of severe lesions (Grade 2: 34.15% vs. 20.90%; Grade 3: 32.93% vs. 12.69%) in the progression group (*p* < 0.001).

The acute infarct volume analysis (Figure 3B) showed that the lesion volume was significantly larger in the progression group compared to the non-progression group [1.30 (0.82–1.98) vs. 0.64 (0.31–1.46) mL, *p* < 0.001]. The quartile analysis of infarct volume revealed that the proportion of patients in the higher quartiles (Quartile 3: 40.24% vs. 15.67%; Quartile 4: 31.71% vs. 20.90%) was significantly higher in the progression group, while the proportion in the lowest quartile was significantly lower (10.98% vs. 33.58%, *p* < 0.001) (Figure 4C).

### 3.4 Multivariable analysis of WMH and infarct volume with early progression

In the multivariable logistic regression analysis (Table 4), each 1 mL increase in WMH volume was associated with a significantly increased risk of early progression (unadjusted OR: 1.01, 95% CI: 1.01–1.02, *p* < 0.001). This association remained significant after adjusting for age, clinical characteristics, and vascular risk factors (fully adjusted OR: 1.01, 95% CI: 1.00–1.02, *p* = 0.047). Quartile analysis showed that patients with the highest WMH burden (fourth quartile) had a significantly increased risk of early progression compared to the first quartile (unadjusted OR: 5.69, 95% CI: 2.42–13.39, *p* < 0.001; fully adjusted OR: 5.32, 95% CI: 1.48–13.88, *p* = 0.009). Trend analysis revealed a significant dose–response relationship between WMH burden and the risk of progression (*P* for trend = 0.005).

**Table 4:** Multivariable logistic regression models for early neurological progression according to WMH burden, Fazekas scale grade, and infarct volume in AChA territory infarction.

Variables	Model 1		Model 2		Model 3	
	OR (95% CI)	*P*-value	OR (95% CI)	*P*-value	OR (95% CI)	*P*-value
WMH volume (per mL increase)	1.01(1.01, 1.02)	0.001	1.01 (1.00, 1.02)	0.030	1.01 (1.00, 1.02)	0.047
Infarct volume (per mL increase)	1.43 (1.14, 1.79)	0.002	1.25 (0.96, 1.61)	0.097	1.18 (0.91, 1.53)	0.217
WMH volume quartiles						
Quartile 1	reference		reference		reference	
Quartile 2	1.65 (0.69, 4.07)	0.269	1.54 (0.59, 4.12)	0.382	1.73 (0.50, 3.67)	0.557
Quartile 3	3.13 (1.36, 7.56)	0.009	2.26 (0.83, 6.34)	0.113	2.44 (0.82, 6.55)	0.118
Quartile 4	5.27 (2.30, 12.81)	<0.001	4.66 (1.60, 14.20)	0.005	5.32 (1.48, 13.88)	0.009
*P* for trend		<0.001		0.004		0.005
Fazekas scale grade						
Grade 0	reference		reference		reference	
Grade 1	1.28 (0.45, 4.18)	0.662	1.52 (0.51, 5.25)	0.473	1.38 (0.45, 4.84)	0.589
Grade 2	4.00 (1.40, 13.38)	0.014	4.60 (1.42, 17.25)	0.015	4.45 (1.34, 17.12)	0.020
Grade 3	6.35 (2.13, 22.07)	0.002	7.26 (2.12, 28.62)	0.003	6.22 (1.74, 25.42)	0.007
*P* for trend		<0.001		<0.001		<0.001
Infarct volume quartiles						
Quartile 1	reference		reference		reference	
Quartile 2	1.75 (0.69, 4.62)	0.243	1.38 (0.50, 3.92)	0.532	1.50 (0.53, 4.23))	0.444
Quartile 3	7.86 (3.30, 20.27)	<0.001	5.42 (2.10, 14.99)	<0.001	5.63 (2.06, 15.40)	<0.001
Quartile 4	4.64 (1.96, 11.84)	<0.001	2.54 (0.86, 7.81)	0.095	2.13 (0.69, 6.59)	0.188
*P* for trend		<0.001		<0.001		<0.001

Values are expressed as odds ratios (ORs) with 95% confidence intervals (CIs). AChA, anterior choroidal artery; WMH, white matter hyperintensities; NIHSS, National Institutes of Health Stroke Scale; mRS, modified Rankin Scale; LDL, low-density lipoprotein. Model 1: Unadjusted. Model 2: Adjusted for age, diabetes mellitus, baseline NIHSS score, baseline mRS score, Anatomical involvement, lesion size. Model 3: Additionally adjusted for laboratory findings (Neutrophils, LDL cholesterol, fibrinogen). WMH quartiles (mL): Q1 [0–5.61), Q2 [5.61–15.9), Q3 [15.9–42), Q4 [42–181.0]. Infarct volume quartiles (mL): Q1 [0–0.387), Q2 [0.387–0.896), Q3 [0.896–1.63), Q4 [1.63–9.14].

The Fazekas scale score analysis showed similar trends. Compared to Grade 0, patients with Grade 3 had a significantly increased risk of early progression (unadjusted OR: 6.35, 95% CI: 2.13–22.07; fully adjusted OR: 8.78, 95% CI: 2.27–40.48, *p* = 0.003). As the Fazekas score increased, the risk of progression showed a significant increasing trend (*P* for trend < 0.001).

In the infarct volume analysis, each 1 mL increase in infarct volume was associated with an increased risk of early progression (unadjusted OR: 1.43, 95% CI: 1.14–1.79, *p* = 0.002), but this association weakened after adjusting for potential confounders (fully adjusted OR: 1.18, 95% CI: 0.91–1.53, *p* = 0.217). Quartile analysis showed that patients in the third quartile had the highest risk of progression compared to the first quartile (fully adjusted OR: 5.63, 95% CI: 2.06–15.40, *p* < 0.001). Trend analysis also showed a significant association between infarct volume and the risk of progression (*P* for trend < 0.001).

### 3.5 Non-linear relationships and threshold effects

**Figure 5:**
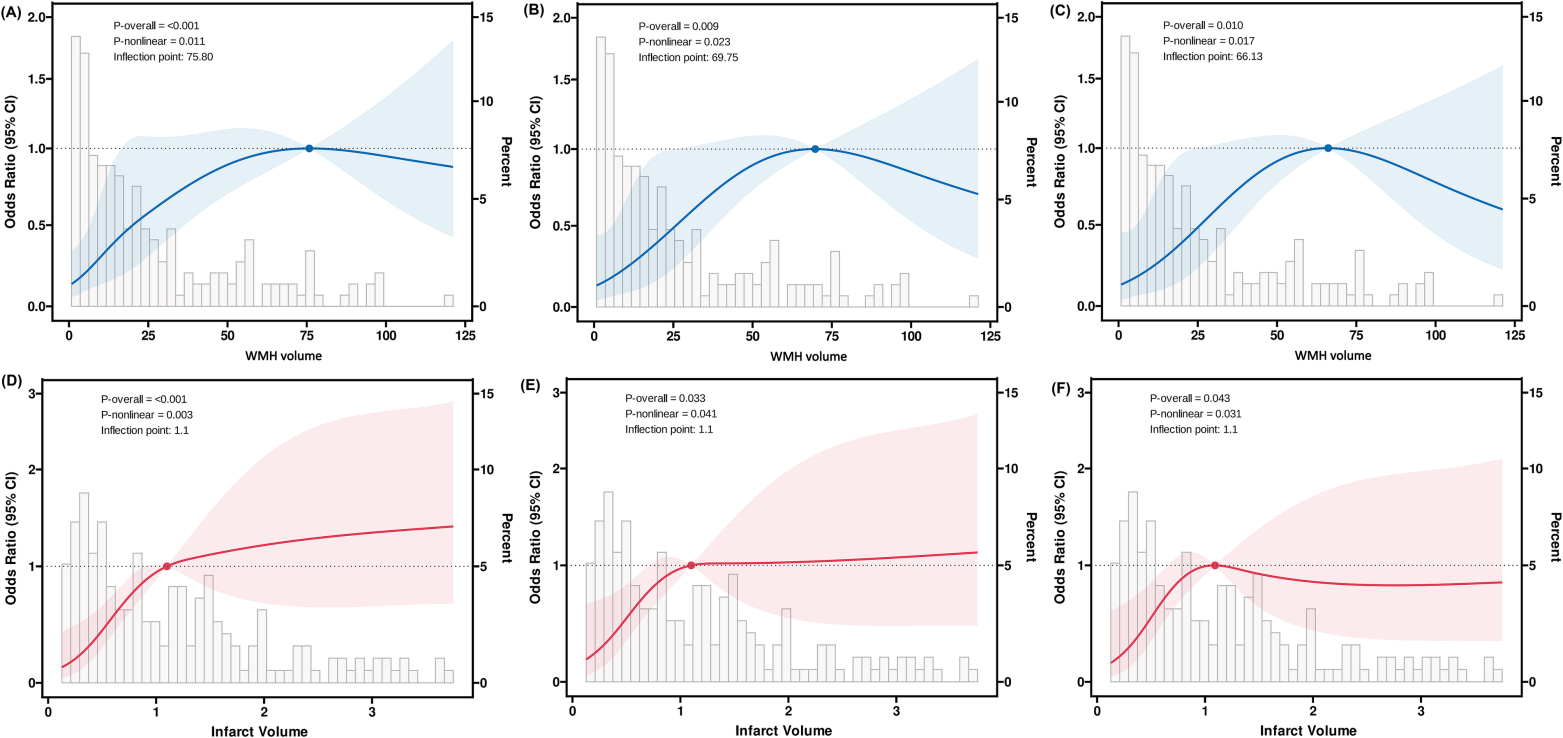
Restricted cubic spline analysis of WMH and infarct volumes with early neurological progression. Restricted cubic spline analysis demonstrating non-linear relationships between imaging markers and early neurological progression. Blue and red lines represent adjusted odds ratios with 95% confidence intervals (shaded areas). Model 1 **(A,D)**: unadjusted; Model 2 **(B,E)**: adjusted for age, clinical characteristics, and anatomical involvement; Model 3 **(C,F)**: additionally adjusted for laboratory findings.

After adjusting for key confounding factors, restricted cubic spline regression analysis revealed significant non-linear associations between WMH volume and infarct volume with early neurological progression (Figure 5 and Table 5). The RCS curve for WMH volume showed a clear inflection point (66.1 mL) and an “inverted U-shaped” distribution (*P*-non-linear = 0.017). Piecewise regression analysis demonstrated that when WMH volume was less than 66.1 mL, each 1 mL increase in WMH volume was significantly associated with the risk of early progression (adjusted OR: 1.03, 95% CI: 1.01–1.05; standardized OR: 1.74, 95% CI: 1.29–2.40; both *p* < 0.001). However, when WMH volume exceeded 66.1 mL, this association significantly weakened (adjusted OR: 1.00, 95% CI: 0.98–1.02; standardized OR: 0.96, 95% CI: 0.46–2.06; both *p* = 0.92).

**Table 5:** Segmented regression analysis of WMH and infarct volume for early neurological progression in AChA territory infarction based on restricted cubic spline-derived thresholds.

Variables	OR (95% CI)	*P*-value	OR per SD (95% CI)	*P*-value
WMH volume				
<66.1 mL	1.03 (1.01–1.05)	<0.001	1.74 (1.29–2.40)	<0.001
≥66.1 mL	1.00 (0.98–1.02)	0.92	0.96 (0.46–2.06)	0.92
Infarct volume				
<1.1 mL	5.50 (1.14–28.1)	0.036	1.59 (1.04–2.47)	0.036
≥1.1 mL	1.01 (0.78–1.31)	0.96	1.01 (0.67–1.54)	0.96

Values are presented as odds ratios (ORs) with 95% confidence intervals (CIs). OR per SD represents the odds ratio per 1-standard deviation increase. All models were adjusted for age, diabetes mellitus, baseline NIHSS score, baseline mRS score, Anatomical involvement, lesion size. Model 3: Additionally adjusted for laboratory findings (Neutrophils, LDL cholesterol, fibrinogen).

Similarly, the relationship between infarct volume and early progression also exhibited a significant non-linear characteristic (*P*-non-linear = 0.043), with an inflection point of 1.1 mL. Within the range of infarct volumes less than 1.1 mL, an increase in infarct volume was significantly associated with the risk of early progression (adjusted OR: 5.50, 95% CI: 1.14–28.1; standardized OR: 1.59, 95% CI: 1.04–2.47; both *p* = 0.036). When the infarct volume exceeded 1.1 mL, this association disappeared (adjusted OR: 1.01, 95% CI: 0.78–1.31; standardized OR: 1.01, 95% CI: 0.67–1.54; both *p* = 0.96).

### 3.6 Subgroup analyses

**Figure 6:**
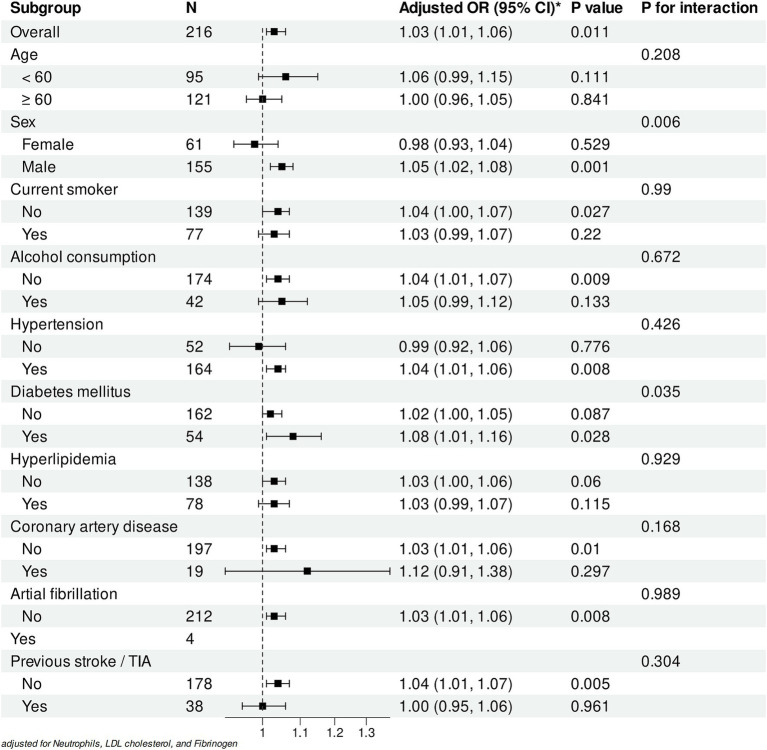
Subgroup analyses of the association between WMH volume and early neurological progression. Forest plot showing adjusted odds ratios (ORs) for the association between white matter hyperintensity (WMH) volume (per 1 mL increase) and early neurological deterioration in different subgroups. All analyses were adjusted for Neutrophils, LDL cholesterol, and Fibrinogen levels.

Subgroup analyses revealed varying associations between WMH volume and early neurological deterioration across different populations (Figure 6). In the overall cohort, each 1-mL increase in WMH volume was significantly associated with increased risk of early progression (adjusted OR: 1.03, 95% CI: 1.01–1.06, *p* = 0.011). A significant sex-specific interaction was observed (*P* for interaction = 0.006), with the association being pronounced in male patients (OR: 1.05, 95% CI: 1.02–1.08, *p* = 0.001) but not significant in female patients (OR: 0.98, 95% CI: 0.93–1.04, *p* = 0.529).

Among vascular risk factors, patients with diabetes mellitus demonstrated a stronger association (OR: 1.08, 95% CI: 1.01–1.16, *p* = 0.028; *P* for interaction = 0.035). Similarly, a significant association was observed in patients with hypertension (OR: 1.04, 95% CI: 1.01–1.06, *p* = 0.008) but not in those without (OR: 0.99, 95% CI: 0.92–1.06, *p* = 0.776).

Analysis of lifestyle factors showed significant associations in non-alcohol consumers (OR: 1.04, 95% CI: 1.01–1.07, *p* = 0.009) and non-smokers (OR: 1.04, 95% CI: 1.00–1.07, *p* = 0.027). Notably, patients without previous stroke/TIA exhibited a stronger association (OR: 1.04, 95% CI: 1.01–1.07, *p* = 0.005), whereas no significant association was found in those with prior stroke/TIA history (OR: 1.00, 95% CI: 0.95–1.06, *p* = 0.961).

Except for sex and diabetes mellitus, no significant interactions were observed across other subgroups (all *P* for interaction > 0.05). All analyses were adjusted for neutrophil count, LDL cholesterol, and fibrinogen levels. These findings suggest that while the association between WMH volume and early progression remains consistent across most populations, differential effects may exist in specific subgroups.

## 4 Discussion

This study is the first to systematically investigate the associations of WMH burden and infarct volume in the AChA territory with early neurological progression. Our study has the following key findings. First, through both quantitative volumetric measurement and Fazekas visual scoring, we confirmed that WMH burden was significantly associated with the risk of early progression, and this association remained robust after adjusting for key confounding factors. Second, WMH burden and the risk of progression showed a significant non-linear relationship, with an inverted U-shaped distribution. When WMH volume was below the threshold (66.1 mL), each one-standard-deviation increase in WMH burden was associated with a 74% increased risk of progression (standardized OR: 1.74, 95% CI: 1.29–2.40, *p* < 0.001). Subgroup analysis showed that the association between WMH burden and early progression had good consistency across major population subgroups.

Our findings are generally consistent with previous literature. Nam et al. (2016) assessed 82 patients with isolated pontine infarction using the Fazekas scale and found that periventricular white matter hyperintensities (OR: 6.17, 95% CI: 1.93–19.75, *p* = 0.002) and deep white matter hyperintensities (OR: 3.19, 95% CI: 1.10–9.23, *p* = 0.032) were both independent predictors of early neurological progression. Wang et al. (2023) evaluated 736 patients with ischemic stroke using the van Swieten scale and similarly confirmed an independent association between white matter lesions and early neurological progression (OR: 1.570, 95% CI: 1.226–2.012). Zhang L. et al. (2024) and Zhang W. et al. (2024) used the Fazekas scale to assess WMH severity, and logistic regression analysis revealed that moderate-to-severe WMHs were an independent predictor of early progression in patients with embolic stroke of undetermined source (OR: 4.012, 95% CI: 1.080–14.906, *p* = 0.038). However, Chen et al. (2017) studied 687 patients with mild-to-moderate acute ischemic stroke and found no significant association between PVWMH or centrum semiovale WMH and early neurological deterioration after adjusting for confounding factors. Song et al. (2024) assessed 107 patients with isolated pontine infarction using the Fazekas scale and did not find an association between imaging markers of cerebral small vessel disease, such as WMHs, lacunes, cerebral microbleeds, and enlarged perivascular spaces, and early progression. Several reasons may account for the differences in research findings: First, the ischemic stroke subtypes included in each study were heterogeneous, suggesting that the relationship between WMHs and early progression may vary depending on the cause and location of the stroke. Second, commonly used WMH visual rating scales (such as the Fazekas scale and van Swieten scale) have a certain degree of subjectivity, and the scoring results may be influenced by the assessor’s experience and judgment criteria, leading to reduced comparability between different studies. Furthermore, visual scoring can hardly comprehensively reflect the heterogeneous spatial distribution characteristics of WMHs, potentially underestimating the true association strength between WMH burden and early neurological regression.

Despite the specific mechanisms underlying the association between WMH burden and early progression not being fully elucidated, we propose several potential mechanistic hypotheses based on existing research evidence. First, WMH burden may reflect an underlying state of diffuse cerebral hypoperfusion. Previous studies have shown that white matter hyperintensity volume is significantly negatively correlated with cerebral blood flow (Shi et al., 2016). A prospective study by ten Dam et al. (2007) further confirmed that a decrease in total cerebral blood flow can predict an increase in PVWMH after 2.75 years. Moreover, multiple studies have found a dose–response relationship between WMHs and poor collateral circulation, with patients with severe WMHs having a significantly higher incidence of collateral circulation dysfunction (Lin et al., 2020; Xu et al., 2022). Therefore, we speculate that patients with high WMH burden may have more severe collateral circulation impairment and global cerebral hypoperfusion, thereby increasing the risk of early neurological progression. This speculation is consistent with the findings of Huang et al. (2022), who used perfusion MRI to assess patients with acute subcortical infarction and found that collateral circulation dysfunction and focal hypoperfusion were significantly associated with early functional deterioration.

Second, WMH burden reflects the chronic accumulation of small vessel lesions, and this long-term damage can lead to a decline in the neural reserve capacity of brain tissue. WMHs may indicate an increased susceptibility of brain tissue to ischemic injury (Huo et al., 2019; Kim and Lee, 2015). At the same time, reduced cerebral blood flow may lead to tissue hypoxia, particularly when the metabolic demand of brain tissue increases. An autopsy study confirmed that WMH regions in the elderly exhibit significant hypoxic injury, as evidenced by the significant upregulation of hypoxia-related molecular markers (such as hypoxia-inducible factor-1α, HF-2α, and matrix metalloproteinase-7) (Fernando et al., 2006). On the basis of pre-existing chronic hypoxic changes and decreased neural reserve capacity, whether acute ischemic events induce more intense oxidative stress responses, thereby more readily leading to neurological progression, is a potential mechanism that warrants further investigation.

Furthermore, several meta-analyses have revealed an association between intracranial and extracranial atherosclerosis and WMH (Fernando et al., 2006; Liu et al., 2024; Zhang W. et al., 2024). Sohn et al. (2013) found that among patients with AChA infarction, the progression group had a significantly higher proportion of severe stenosis or occlusion of the ipsilateral internal carotid artery compared to the non-progression group, suggesting that progressive AChA infarction may be closely related to local large artery lesions. The potential mechanism may be as follows: large artery stenosis leads to reduced distal perfusion pressure and decreased microemboli clearance rate, which in turn causes microemboli retention and ultimately exacerbates neurological deficits. However, due to the lack of large vessel assessment data in this study, we were unable to further explore the possible mediating effects between carotid artery stenosis, WMHs, and early progression. This mechanism requires validation in future studies.

This study found that the proportion of large lesions (maximum diameter ≥20 mm) was significantly higher in the progression group than in the non-progression group. Trend analysis also showed a significant association between infarct volume and the risk of progression. This result is consistent with previous research. Ois et al. (2009) analyzed 112 patients with AChA infarction and found that the proportion of progression was much higher in the large infarct group (≥20 mm) than in the small infarct group (38% vs. 17.1%, *p* = 0.013). Notably, our study revealed a non-linear relationship between infarct volume and the risk of early neurological progression through quantitative analysis. After fully adjusting for confounding factors, patients with infarct volumes in the third quartile range exhibited the highest risk of progression (adjusted OR: 5.63, 95% CI: 2.06–15.40, *p* < 0.001). Further analysis showed that this association had a significant non-linear characteristic and a critical volume threshold (1.1 mL). When the infarct volume was below this threshold, each one-standard-deviation increase was associated with a 59% increased risk of progression (standardized OR: 1.59, 95% CI: 1.04–2.47, *p* = 0.036). This complex association pattern may reflect the heterogeneous pathogenesis of AChA infarction.

Previous studies have found that, compared to other deep small infarcts, AChA infarction is more closely associated with severe carotid artery stenosis and cardiogenic embolism (Hupperts et al., 1994). Sohn et al. (2013) analyzed the DWI and MRA findings of 127 patients with AChA infarction and suggested that small vessel lesions caused by large artery atherosclerosis may be the main cause of AChA infarction. Ois et al.’s (2009) study also reached similar conclusions, speculating that small infarcts may originate from perforating artery occlusion, while large infarcts may be caused by cardiogenic emboli or complete AChA occlusion due to large artery lesions. We speculate that perforating artery occlusion is more likely to cause continuous neurological progression, while large infarct lesions caused by embolic mechanisms may reach the maximum degree of neurological deficits at the onset of the disease, and thus have a relatively lower probability of further progression. However, current studies are mostly limited to assessing the association between infarct size and early progression using maximum lesion diameter, lacking precise quantitative analysis of infarct volume. Therefore, the finding of a non-linear association between infarct volume and progression risk in this study still requires further validation in larger prospective studies.

Our study has several notable limitations. As a retrospective investigation, selection and information biases were unavoidable. Additionally, only a small proportion of patients (26.39%) underwent comprehensive head and neck CTA examination. Although we found no statistically significant differences in vascular stenosis between the progression and non-progression groups, this finding may be limited by insufficient sample size. Due to the constraints in vascular imaging data, we were unable to thoroughly explore the potentially complex associations between large vessel disease, collateral circulation status, WMH burden, and early neurological progression. Furthermore, the imaging prediction thresholds identified in this study require external validation through large-scale, multicenter prospective studies to establish their clinical utility. Based on these limitations, we propose the following recommendations for future research: First, conducting standardized prospective cohort studies to not only validate the imaging predictive thresholds identified in our study but also systematically evaluate their predictive value for long-term patient outcomes. Second, employing advanced image segmentation techniques for precise quantitative analysis of deep and periventricular white matter hyperintensities, and integrating multimodal imaging techniques such as diffusion tensor imaging to investigate the specific associations between microstructural changes in different WMH regions and progression risk. Third, expanding the sample size and performing quantitative objective analysis of vascular stenosis, considering mediation effect analysis to explore the intrinsic connections between large vessel disease, WMH burden, and early progression. Finally, integrating blood biomarker analysis to explore the intrinsic associations between inflammatory factors, oxidative stress indicators, WMH burden, and early progression, with the aim of further elucidating the underlying pathophysiological mechanisms.

## 5 Conclusion

This study is the first to systematically investigate the associations of WMH burden and infarct volume in the AChA territory with early neurological progression. The study found that WMH burden had a significant non-linear association with the risk of early progression and successfully identified predictive thresholds with potential clinical application value (WMH volume 66.1 mL, infarct volume 1.1 mL). These findings not only deepen the understanding of the mechanisms underlying early progression in AChA territory infarction but also provide new insights for clinical practice: early identification of high-risk patients through magnetic resonance imaging markers can help formulate more targeted monitoring and intervention strategies.

## Data Availability

The datasets presented in this article are not readily available because the datasets used and/or analyzed during the current study are available from the corresponding author upon reasonable request. Requests to access the datasets should be directed to Renjing Zhu, zhurenjing@163.com.

## References

[ref1] Antoniello D. Patel N. R. Ladsaria S. S. (2023). Three-dimensional maps of the anterior choroidal artery territory. Stroke 54, e73–e74. doi: 10.1161/STROKEAHA.122.042011, PMID: 36655555

[ref2] Chausson N. Joux J. Saint-Vil M. Edimonana M. Jeannin S. Aveillan M. (2014). Infarction in the anterior choroidal artery territory: clinical progression and prognosis factors. J. Stroke Cerebrovasc. Dis. 23, 2012–2017. doi: 10.1016/j.jstrokecerebrovasdis.2014.02.013, PMID: 25088169

[ref3] Chen Z. Li W. Sun W. Xiao Z. Zhao Y. Zhao J. (2017). Correlation study between small vessel disease and early neurological deterioration in patients with mild/moderate acute ischemic stroke. Int. J. Neurosci. 127, 579–585. doi: 10.1080/00207454.2016.1214825, PMID: 27430627

[ref4] Deguchi I. Takahashi S. (2023). Pathophysiology and optimal treatment of intracranial branch atheromatous disease. J. Atheroscler. Thromb. 30, 701–709. doi: 10.5551/jat.RV22003, PMID: 37183021 PMC10322737

[ref5] Derflinger S. Fiebach J. B. Böttger S. Haberl R. L. Audebert H. J. (2015). The progressive course of neurological symptoms in anterior choroidal artery infarcts. Int. J. Stroke 10, 134–137. doi: 10.1111/j.1747-4949.2012.00953.x, PMID: 23294991

[ref6] Fernando M. S. Simpson J. E. Matthews F. Brayne C. Lewis C. E. Barber R.. (2006). White matter lesions in an unselected cohort of the elderly: molecular pathology suggests origin from chronic hypoperfusion injury. Stroke 37, 1391–1398. doi: 10.1161/01.STR.0000221308.94473.14, PMID: 16627790

[ref7] Herman L. H. Fernando O. U. Gurdjian E. S. (1966). The anterior choroidal artery: an anatomical study of its area of distribution. Anat. Rec. 154, 95–101. doi: 10.1002/ar.1091540109, PMID: 5922500

[ref8] Huang Y. C. Lee J. D. Pan Y. T. Chen Y. W. Tseng Y. C. Lin S. K.. (2022). Perfusion defects and collateral flow patterns in acute small subcortical infarction: a 4D dynamic MRI study. Transl. Stroke Res. 13, 399–409. doi: 10.1007/s12975-021-00953-x, PMID: 34648143 PMC9046333

[ref9] Huang W. Q. Lin Q. Tzeng C. M. (2024). Leukoaraiosis: epidemiology, imaging, risk factors, and management of age-related cerebral white matter hyperintensities. J Stroke 26, 131–163. doi: 10.5853/jos.2023.02719, PMID: 38836265 PMC11164597

[ref10] Huo Y. C. Li Q. Zhang W. Y. Zou N. Li R. Huang S. J.. (2019). Total small vessel disease burden predicts functional outcome in patients with acute ischemic stroke. Front. Neurol. 10:808. doi: 10.3389/fneur.2019.00808, PMID: 31447754 PMC6691043

[ref11] Hupperts R. M. Lodder J. Heuts-van Raak E. P. Kessels F. (1994). Infarcts in the anterior choroidal artery territory: anatomical distribution, clinical syndromes, presumed pathogenesis and early outcome. Brain 117, 825–834. doi: 10.1093/brain/117.4.825, PMID: 7922468

[ref12] Kim B. J. Lee S. H. (2015). Prognostic impact of cerebral small vessel disease on stroke outcome. J Stroke 17, 101–110. doi: 10.5853/jos.2015.17.2.101, PMID: 26060797 PMC4460329

[ref13] Lin M. P. Brott T. G. Liebeskind D. S. Meschia J. F. Bath P. M. Yoo A. J. (2020). Collateral recruitment is impaired by cerebral small vessel disease. Stroke 51, 1404–1410. doi: 10.1161/STROKEAHA.119.027661, PMID: 32248770

[ref14] Liu H. Liu K. Zhang K. Sun W. Liu Y. Qi P.. (2023). Early neurological deterioration in patients with acute ischemic stroke: a prospective multicenter cohort study. Ther. Adv. Neurol. Disord. 16:17562864221147743. doi: 10.1177/17562864221147743, PMID: 36710721 PMC9880581

[ref15] Liu X. Wu N. Li J. Li Y. Wang X. Wang Y. (2024). Risk factors and clinical characteristics of first-ever ischemic stroke caused by ICAS with leukoaraiosis. Int. J. Med. Sci. 21, 1500–1510. doi: 10.7150/ijms.95984, PMID: 38903919 PMC11186426

[ref16] Nam K. W. Lim J. S. Kang D. W. Lee Y. S. Kim S. Kwon H. M. (2016). Severe white matter hyperintensity is associated with early neurological deterioration in patients with isolated pontine infarction. Eur. Neurol. 76, 117–122. doi: 10.1159/000448888 27532619

[ref17] Ois A. Cuadrado-Godia E. Solano A. Perich-Alsina X. Roquer J. (2009). Acute ischemic stroke in anterior choroidal artery territory. J. Neurol. Sci. 281, 80–84. doi: 10.1016/j.jns.2009.02.323 19324377

[ref18] Seners P. Turc G. Oppenheim C. Baron J. C. (2015). Incidence, causes and predictors of neurological deterioration occurring within 24 h following acute ischaemic stroke: a systematic review with pathophysiological implications. J. Neurol. Neurosurg. Psychiatry 86, 87–94. doi: 10.1136/jnnp-2014-308327, PMID: 24970907

[ref19] Shi Y. Thrippleton M. J. Makin S. D. Marshall I. Geerlings M. I. de Craen A. J.. (2016). Cerebral blood flow in small vessel disease: a systematic review and meta-analysis. J. Cereb. Blood Flow Metab. 36, 1653–1667. doi: 10.1177/0271678X16662891 27496552 PMC5076792

[ref20] Shkirkova K. Saver J. L. Starkman S. Wong G. Weng J. Hamilton S.. (2018). FAST-MAG trial coordinators and investigators. Frequency, predictors, and outcomes of prehospital and early Postarrival neurological deterioration in acute stroke: exploratory analysis of the FAST-MAG randomized clinical trial. JAMA Neurol. 75, 1364–1374. doi: 10.1001/jamaneurol.2018.1893, PMID: 30039165 PMC6248118

[ref21] Sohn H. Kang D. W. Kwon S. U. Kim J. S. (2013). Anterior choroidal artery territory infarction: lesions confined to versus beyond the internal capsule. Cerebrovasc. Dis. 35, 228–234. doi: 10.1159/000347069, PMID: 23548698

[ref22] Song X. He F. Hou D. Li Y. Chen H. Liu Y.. (2024). Imaging biomarker associated with early neurological deterioration in isolated pontine infarction. Front. Neurol. 15:1492166. doi: 10.3389/fneur.2024.1492166, PMID: 39748858 PMC11694510

[ref23] ten Dam V. H. van den Heuvel D. M. de Craen A. J. Bollen E. L. Murray H. M. Westendorp R. G.. (2007). Decline in total cerebral blood flow is linked with increase in periventricular but not deep white matter hyperintensities. Radiology 243, 198–203. doi: 10.1148/radiol.2431052111, PMID: 17329688

[ref24] Wang D. Yan D. Yan M. Zhang J. Zhang X. Wang Z. (2023). Leukoaraiosis severity is related to increased risk of early neurological deterioration in acute ischemic stroke: a retrospective observational study. Acta Neurol. Belg. 123, 1413–1420. doi: 10.1007/s13760-023-02249-3, PMID: 37014516

[ref25] Wu X. Ya J. Zhou D. Yang Y. Li S. Lu Y.. (2021). Pathogeneses and imaging features of cerebral white matter lesions of vascular origins. Aging Dis. 12, 2031–2051. doi: 10.14336/AD.2021.0414, PMID: 34881084 PMC8612616

[ref26] Xu M. Guo W. Rascle L. Gurram K. Mossa-Basha M. Patel S.. (2022). Leukoaraiosis distribution and cerebral collaterals: a systematic review and meta-analysis. Front. Neurol. 13:869329. doi: 10.3389/fneur.2022.869329, PMID: 35812112 PMC9263359

[ref27] Yang H. Liu H. Zhang K. Liu Y. Sun W. Huang Y.. (2023). Neuroimaging markers of early neurological deterioration in acute isolated pontine infarction. Neurol. Sci. 44, 3607–3614. doi: 10.1007/s10072-023-06837-2, PMID: 37246178

[ref28] Zhang W. Fu F. Zhan Z. (2024). Association between intracranial and extracranial atherosclerosis and white matter hyperintensities: a systematic review and meta-analysis. Front. Aging Neurosci. 15:1240509. doi: 10.3389/fnagi.2023.1240509, PMID: 38259641 PMC10800362

[ref29] Zhang L. Su Y. Wang Q. Zhang Y. Wu J. Li Y. (2024). Predictive value of white matter hyperintensities for early neurological deterioration in patients with embolic stroke of undetermined source. Neuropsychiatr. Dis. Treat. 20, 2049–2055. doi: 10.2147/NDT.S472626, PMID: 39494382 PMC11531716

[ref30] Zhao N. Li J. Zhang Q. X. Ding Y. C. Wu J. H. Sun X. Y. (2023). Elevated neutrophil-related immune-inflammatory biomarkers in acute anterior choroidal artery territory infarction with early progression. Clin. Neurol. Neurosurg. 229:107720. doi: 10.1016/j.clineuro.2023.107720 37084652

[ref31] Zhou Z. Malavera A. Yoshimura S. Lambrou D. Goyal N. Gonzalez R. G.. (2020). Clinical prognosis of FLAIR hyperintense arteries in ischaemic stroke patients: a systematic review and meta-analysis. J. Neurol. Neurosurg. Psychiatry 91, 475–482. doi: 10.1136/jnnp-2019-322625, PMID: 32217786

