# Supplementary material for: Association of white matter hyperintensity burden and infarct volume in the anterior choroidal artery territory with early neurological progression: a dual-center retrospective study
Source: Front Aging Neurosci. 2025 May 19;17:1577742. doi: 10.3389/fnagi.2025.1577742 (PMC12127335; doi:10.3389/fnagi.2025.1577742)
Supplement: Supplementary file 1 [file Data_Sheet_1.docx]

| **Supplemental Table 1.** Multicollinearity Assessment of Variables Included in the Multivariable Logistic Regression Models | | | | |
| --- | --- | --- | --- | --- |
| **Variable** | **VIF*** | **Tolerance** | **VIF**** | **Tolerance**** |
| WMH volume | 1.210 | 0.826 | - | - |
| Infarct Volume | - | - | 1.397 | 0.715 |
| Age, years | 1.317 | 0.759 | 1.128 | 0.886 |
| Diabetes mellitus | 1.039 | 0.962 | 1.041 | 0.960 |
| Baseline NIHSS score | 3.131 | 0.319 | 3.287 | 0.304 |
| Baseline mRS score | 3.244 | 0.308 | 3.333 | 0.300 |
| Lesion size | 1.076 | 0.929 | 1.328 | 0.753 |
| Anatomical involvement |  |  |  |  |
| Lateral thalamus | 1.024 | 0.977 | 1.025 | 0.976 |
| Lateral geniculate body | 1.000 | 0.999 | 1.000 | 0.999 |
| Medial temporal lobe / hippocampus | 1.040 | 0.962 | 1.038 | 0.963 |
| Neutrophil count | 1.102 | 0.907 | 1.117 | 0.895 |
| LDL cholesterol | 1.126 | 0.888 | 1.154 | 0.867 |
| Fibrinogen | 1.073 | 0.932 | 1.090 | 0.918 |
| Variance inflation factor (VIF) values <10 and tolerance values >0.1 indicate acceptable multicollinearity levels. **Abbreviations:** NIHSS, National Institutes of Health Stroke Scale; mRS, modified Rankin Scale; LDL, low-density lipoprotein; WMH, white matter hyperintensities. | | | | |
